# Segmentation in dermatological hyperspectral images: dedicated methods

**DOI:** 10.1186/s12938-016-0219-5

**Published:** 2016-08-17

**Authors:** Robert Koprowski, Paweł Olczyk

**Affiliations:** 1 Department of Biomedical Computer Systems, University of Silesia, Bedzinska 39, 41-200 Sosnowiec, Poland; 2 Department of Community Pharmacy, School of Pharmacy and Division of Laboratory Medicine in Sosnowiec, Medical University of Silesia in Katowice, Kasztanowa 3, 41-200 Sosnowiec, Poland

**Keywords:** Fast segmentation method, Image processing, Thresholding, Conditional erosion, Conditional dilatation, Hyperspectral imaging

## Abstract

**Background:**

Segmentation of hyperspectral medical images is one of many image segmentation methods which require profiling. This profiling involves either the adjustment of existing, known image segmentation methods or a proposal of new dedicated methods of hyperspectral image segmentation. Taking into consideration the size of analysed data, the time of analysis is of major importance. Therefore, the authors proposed three new dedicated methods of hyperspectral image segmentation with special reference to the time of analysis.

**Methods:**

The segmentation methods presented in this paper were tested and profiled to the images acquired from different hyperspectral cameras including SOC710 Hyperspectral Imaging System, Specim sCMOS-50-V10E. Correct functioning of the method was tested for over 10,000 2D images constituting the sequence of over 700 registrations of the areas of the left and right hand and the forearm.

**Results:**

As a result, three new methods of hyperspectral image segmentation have been proposed: fast analysis of emissivity curves (SKE), 3D segmentation (S3D) and hierarchical segmentation (SH). They have the following features: are fully automatic; allow for implementation of fast segmentation methods; are profiled to hyperspectral image segmentation; use emissivity curves in the model form, can be applied in any type of objects not necessarily biological ones, are faster (SKE—2.3 ms, S3D—1949 ms, SH—844 ms for the computer with Intel^®^ Core i7 4960X CPU 3.6 GHz) and more accurate (SKE—accuracy 79 %, S3D—90 %, SH—92 %) in comparison with typical methods known from the literature.

**Conclusions:**

Profiling and/or proposing new methods of hyperspectral image segmentation is an indispensable element of developing software. This ensures speed, repeatability and low sensitivity of the algorithm to changing parameters.

## Background

Image segmentation is the process of dividing an image into parts defined as areas which are homogeneous in terms of selected properties. Today, segmentation is one of the most widely used [1, 2] and overused word in the area of biomedical image analysis (PubMed database contains 23,202 results—using the search strategy “all fields”, scopus—150,147 results—using the search strategy “all fields”). Practically everywhere where a region of interest (ROI) is separated using any method, the authors call it segmentation [3, 4]. Of course, in some cases, this is justified [5]. Formally, however, segmentation of images is divided into four main groups [6–12]:

- point operations,
- edge methods,
- region methods and
- hybrid methods.

Today, the most developed are region methods which include [13–16]:

- region growing,
- region merging,
- region splitting,
- split and merge,
- watershed segmentation.

**Fig. 1:**
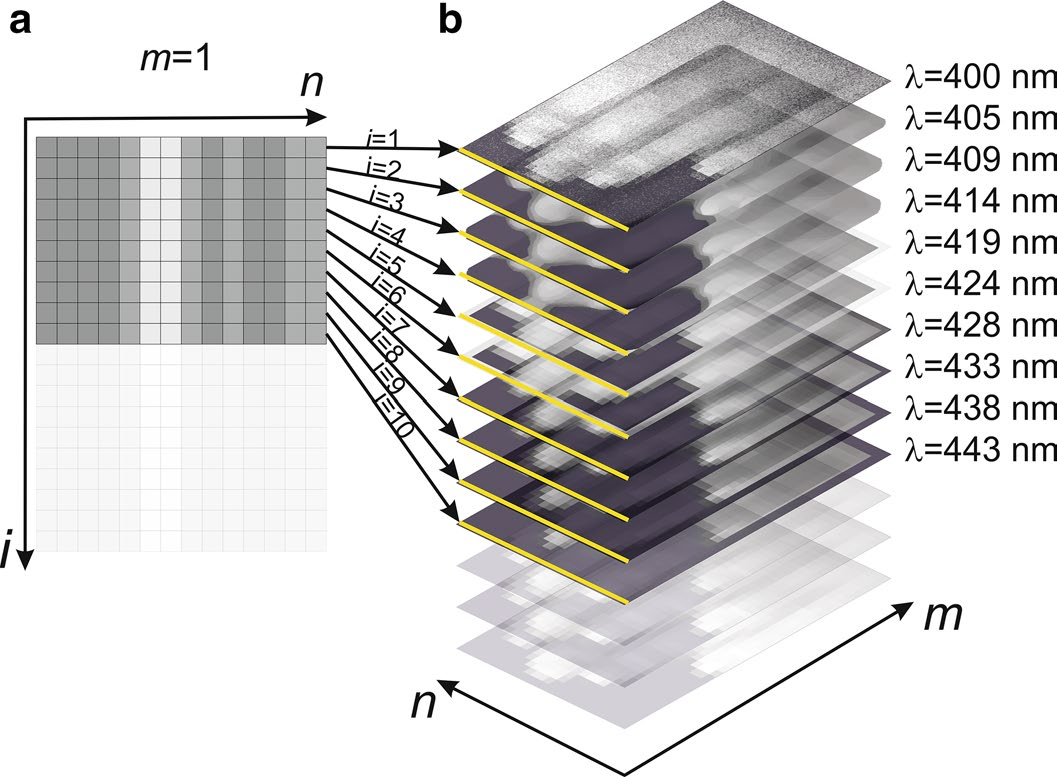
Illustrative system of data organization in *.raw, *.dat and *.cube (files obtained from a hyperspectral camera) and the result of conversion to a sequence of 2D images

Not all segmentation methods can be applied in the analysis of medical hyperspectral images [17]. The point methods based on the selection of an appropriate binarization threshold are the most common. The watershed and hybrid methods are equally popular. Their application is limited due to two main elements. The first one is segmentation of an object whose limit, contour, is a change in the brightness of pixels. It is most often calculated for one or three (RGB) brightness levels. The second element is the lack of dependence of segmentation on the reference (expected) spectral spectrum. Therefore, in comparison to the segmentation of images in grey levels or colour images, hyperspectral imaging gives much more opportunities [18]. However, this excess of data is not, in every case, used by the authors of the algorithm (the authors of the article). The main limitation is the above-mentioned lack of methods dedicated for hyperspectral image segmentation. This type of dedicated methods should use, apart from the conventional 2D image analysis, spatial information for individual wavelengths. One of the significant factors facilitating this type of analysis is organization of data written to *.raw, *.dat or *.cube files by a hyperspectral camera. Typical organization of this type of data is shown in Fig. 1.

The data are saved in a file by a hyperspectral camera (Fig. 1a) sequentially, starting with the full spectral range for the first (*m*-th) row of the matrix. In the next steps, data for subsequent rows of the matrix of the image *L*_*GRAY*_(*m*, *n*, *i*) are saved in the file. Therefore segmentation methods can be easily implemented (minimizing computational complexity) using information about the spectral amplitude values stored for each wavelength for one pixel [19, 20]. In the stored *.raw, *.dat or *.cube file this is one (*n*-th) column (see Fig. 1a). In terms of data organization in a file, access to these data is at the beginning (Fig. 1a, b). This type of data organization has a significant influence on the access time. Moreover, an additionally favorable element is the organization of particular first rows of each image consecutively as the first read data from *.raw, *.dat or *.cube files. This specific data organization and specificity of hyperspectral imaging were further used in the dedicated methods of hyperspectral image segmentation presented in the following sections.

## Materials

The presented segmentation methods are related to the images acquired from different hyperspectral cameras (SOC710 Hyperspectral Imaging System, Specim sCMOS-50-V10E). In total, more than 10,000 2D images were acquired, constituting a sequence of more than 700 registrations, for 20 healthy subjects (40 % of women) aged 20–55 years. The test area was the area of the left and right hand as well as the forearm. The subjects expressed their free and informed consent for the study. The images were acquired in accordance with the Helsinki Declaration. Data were obtained retrospectively and no measurements or tests were carried out on the subjects as part of this work. This work only describes new techniques for image analysis. The discussed methods were tested on hyperspectral images from public databases and images described in the authors’ earlier works—for example [5]. The processing methods presented in this article were tested on a computer with Intel^®^ Core i7 4960X CPU 3.6 GHz. The resolution of acquired images *L*_*GRAY*_(*m*, *n*, *i*), where *m*-row, *n*-column, *i*-frame number, was standardized to *M* × *N* × *I* = 696 × 520 × 128 pixels (where *M*-number of rows, *N*-number of columns, *I*-number of bands). The spectral range for 128 bands was from 0.4 to 1.0 µm, dynamic range 12 bit, line rate 33 lines/s, pixels per line 520. The test subjects were illuminated by means of halogen lamps with a power of 100 W and linear radiation in the range of the camera operation.

## Methods

The new dedicated methods of hyperspectral image segmentation were divided into 3 different areas (methods):

- fast analysis of emissivity curves (absorption) of an object,
- 3D segmentation,
- hierarchical segmentation.

The methods were preceded by image pre-processing.

### Image pre-processing

These methods were preceded by image pre-processing. It involved median filtering of the image *L*_*GRAY*_(*m*, *n*, *i*) with a mask sized *M*_*h*_ × *N*_*h*_ × *I*_*h*_ = 3 × 3 × 3 pixels. The size of the filter mask was selected based on the maximum size of a single artefact which was not higher than 4 pixels. The resulting image *L*_*MED*_(*m*, *n*, *i*) was further calibrated on the basis of the calibration bar which, at each registration, was located in the upper part of the image [5], or on the basis of the recorded images [dark *L*_*DARK*_(*m*, *n*, *i*) and white *L*_*WHITE*_(*m*, *n*, *i*)] allowing for conventional calibration and normalization of the image. The resulting image after this pre-processing of the image *L*_*C*_(*m*, *n*, *i*) was the basis for further transformations.

It should be emphasized here that median filtering for the fixed mask size is one of the simplest filtration methods. In a more developed form, median filtering should be adaptive. The necessity of applying the adaptive method is caused by the imaging character of hyperspectral cameras. For each camera, 2D images acquired for threshold wavelength values have the biggest noise [21]. Therefore, one of the possibilities is the application of an adaptive filtration method based on enlarging the mask size of the median filter for 2D images at the band edge. As it was shown in [21], it is the mask size changed depending on the wavelength in the range from *M*_*h*_ × *N*_*h*_ = 7 × 7 pixels to *M*_*h*_ × *N*_*h*_ = 3 × 3 pixels. In extreme cases when there is no information which 2D image will be analysed, the following mask size can be adopted: *M*_*h*_ × *N*_*h*_ = 7 × 7 pixels, taking into account the necessary removal of some minor details for median 2D images (for median wavelengths) [22–26].

### Fast analysis of emissivity curves

According to the authors, the analysis of emissivity curves should be the most commonly used technique of hyperspectral image segmentation. Due to the specific data organisation, it can be used even when reading the beginning of the data in the *.raw, *.dat and *.cube file. Therefore, it can be used for the rough, screening test of compliance of spectrum amplitudes with the model (the expected waveform). The practical realization of such segmentation relates to the analysis of the first row of the matrix *L*_*GRAY*_(*m* = 1, *n*, *i*) or, after image pre-processing, the matrix *L*_*C*_(*m*, *n*, *i*). Assuming that the reference waveform is *L*_*PAT*_(*i*), the analysis for the first column *n* = 1 and the subsequent ones was formulated as:

1 $$L_{D} \left( {m,\;n = 1,\;i} \right) = \frac{{L_{c} \left( {m,\;n = 1,i} \right) - L_{PAT} \left( i \right)}}{{L_{PAT} \left( i \right)}}$$

For the adopted tolerance threshold *p*_*r*_ of the difference between the reference and measured emissivity, the number of individual wavelengths *λ* (number *i*) for which the values *L*_*D*_(*m*, *n*, *i*) exceeded the adopted threshold *p*_*r*_ were calculated—the obtained results constitute a new matrix *L*_*R*_(*m*, *n*), i.e.:

2 $$L_{R} \left( {m,\;n} \right) = \mathop \sum \limits_{i = 1}^{I} L_{BR} \left( {m,\;n,\;i} \right)$$

where:

3 $$L_{BR} \left( {m,\;n,\;i} \right) = \left\{ {\begin{array}{*{20}l} 1 \quad {\text{if}} \, {L_{D} \left( {m,\;n,\;i} \right) < p_{r} } \\ 0 \quad {\text{other}} & \\ \end{array} } \right.$$

Depending on the needs (speed of calculations), this analysis can be narrowed down to the afore-mentioned wavelength values *i* at *m* = 1 and *i* = 1. Table 1 shows the summary of mean analysis times for different values of *m* and *i* for Intel^®^ Core i7 4960X CPU 3.6 GHz. As shown in Table 1, the mean analysis time depends largely on the value of *m*. It is the result of time needed to read the information from the data files, *.raw, *.dat or *.cube (see Fig. 1a). The highest values are obtained at the first reading, and they are equal to 2.3 ms. This increased time results from getting access to data on the disk (in the tested operating system Windows 7 Professional). The time of reading other data for the next values of *m* and *i* increases linearly and for *i*∈(1, 100) and *m* = 100, it is 1.6 ms. For comparison, the time of reading any image *L*_*GRAY*_(*m*, *n*, *i*) for *i* = 1 or *i* = 100 is a minimum of 10 ms.

**Table 1:** Summary of mean analysis times for different values of *m* and *i* for Intel^®^ Core i7 4960X CPU 3.6 GHz [the values are given in (ms)]

	*m* = 1	*m* = 2	*m* = 4	*m* = 10	*m* = 100
*i* = 1	2.3	0.49	0.46	0.53	1.6
*i* ∈ (1,2)	2.2	0.51	0.46	0.55	1.6
*i* ∈ (1,4)	2.3	0.50	0.47	0.53	1.6
*i* ∈ (1,10)	2.3	0.51	0.47	0.56	1.7
*i* ∈ (1,100)	2.3	0.51	0.47	0.55	1.6

The sum of the difference values between the reference and measured waveforms *L*_*R*_(*m*, *n*) is the basis for changing the colour space and, thus, segmentation. The image *L*_*V*_(*m*, *n*), as defined below, is reliable for the grey levels:

4 $$L_{V} \left( {m,\;n} \right) = 1 - \frac{{\left| {L_{R} \left( {m,\;n} \right)} \right|}}{{\mathop {\hbox{max} }\limits_{m,n} \left| {L_{R} \left( {m,\;n} \right)} \right|}}$$

**Fig. 2:**
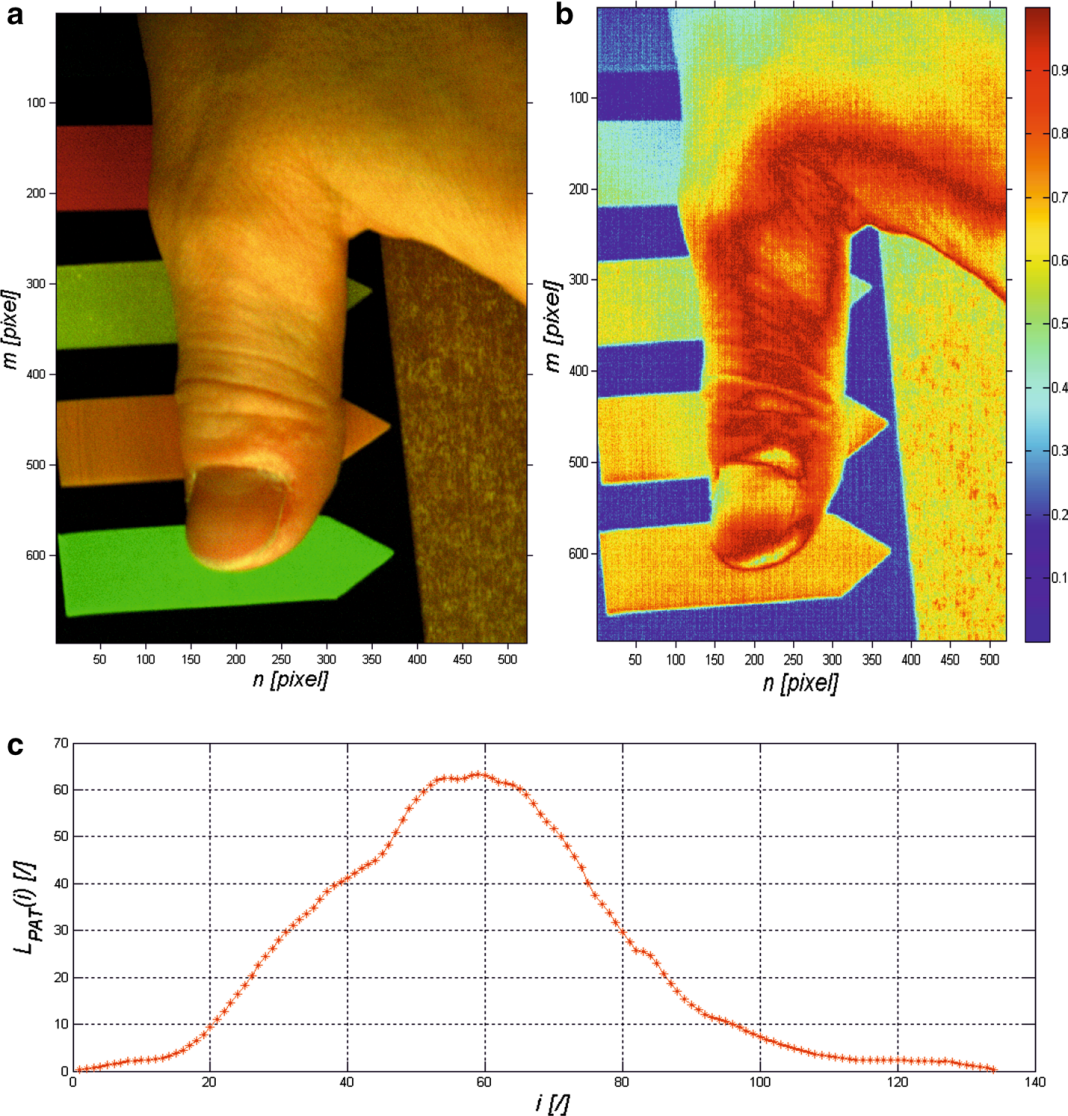
Sample results obtained for the image of a healthy thumb: **a** colour image created on the basis of 3 matrices *L*_*GRAY*_ (520, 450 and 650 nm); **b** matrix *L*_*V*_(*m*, *n*) for the artificial colour palette; **c** reference waveform *L*_*PAT*_(*i*)

**Fig. 3:**
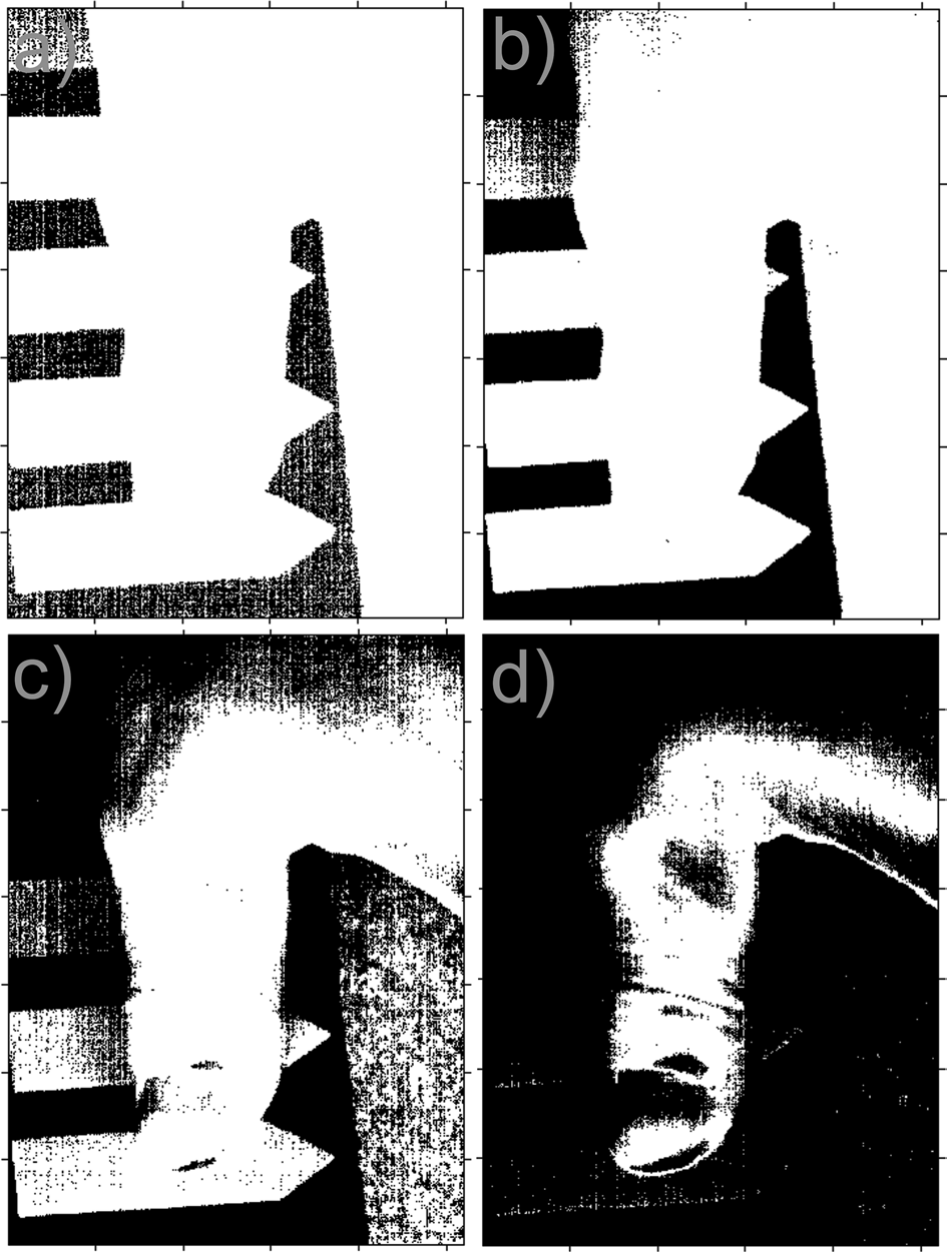
Sample results of segmentation (binarization) for a fast method of segmentation for binarization thresholds of images *L*_*V*_(*m, n*) equal to: **a** 0.2; **b** 0.4; **c** 0.6 and **d** 0.8

In this case, interpretation of the results obtained is intuitive. The value equal to “0” is the maximum value of the error *L*_*V*_(*m, n*), the values close to “1” are the minimum error values. A sample image *L*_*V*_(*m, n*) is shown in Fig. 2. Segmentation of an object (objects) present in a scene can be performed based on the matrix *L*_*V*_(*m, n*) or *L*_*R*_(*m, n*). Sample segmentation results for various binarization thresholds (values 0.2, 0.4, 0.6 and 0.8) were presented in Fig. 3. It should be underlined that the obtained result is for the segmentation based on the first row of the matrix of 2D images for specific wavelengths, while the time of segmentation for the processor Intel^®^ Core i7 4960X CPU 3.6 GHz did not exceed 2.3 ms.

### 3D segmentation

A different approach to segmentation is the analysis of the whole sequence of *i* images *L*_*C*_(*m*, *n*, *i*). For this purpose, the ROI containing a fragment of the segmented object is marked either manually or automatically in the image *L*_*C*_(*m*, *n*, *i*). On this basis, the waveform *L*_*PAT*_(*i*) is created as:

5 $$L_{PAT} \left( i \right) = \frac{1}{{M_{R} \cdot N_{R} }}\mathop \sum \limits_{m,n \in ROI}^{ } L_{C} \left( {m,\;n,\;i} \right)$$

where: *M*_*R*_ and *N*_*R*_—number of rows and columns of the ROI respectively. The image *L*_*V*_(*m*, *n*) is calculated in the next step:

6 $$L_{V} \left( {m,\;n} \right) = L_{C} \left( {m,\;n,\;i} \right) - L_{PAT} \left( i \right)$$

**Fig. 4:**
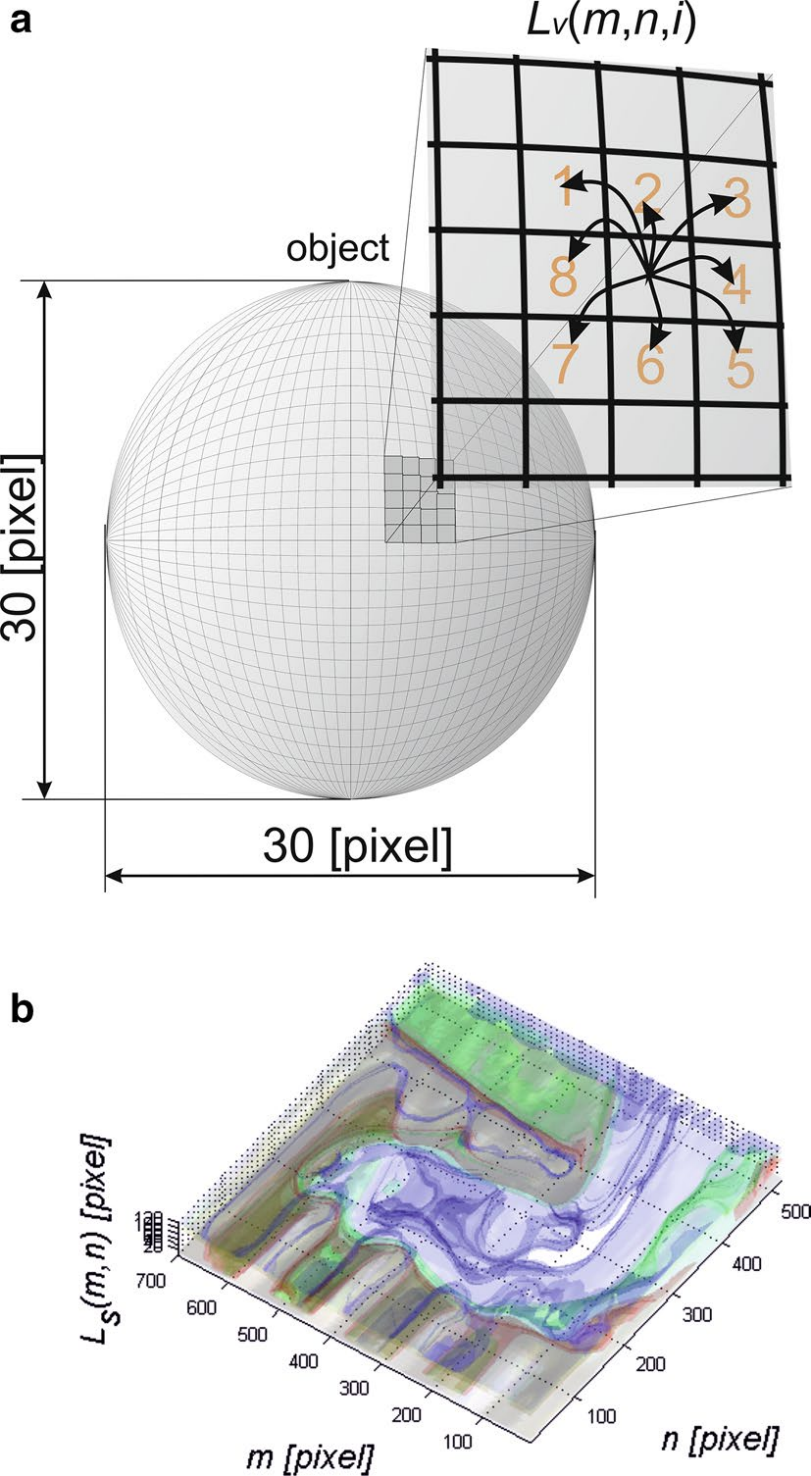
Schematic diagram of filtration for the 8-neighbourhood system: **a** location of a sample pixel of the matrix *L*_*V*_(*m*, *n*, *i*); **b** sample results, image *L*_*S*_(*m*, *n*) for the image of a healthy thumb. *Red*, *green* and *blue* indicate the results of thresholding of the image *L*_*S*_(*m*, *n*) with the thresholds *p*_*r1*_ = 30 %*, p*_*r2*_ = 20 % and *p*_*r3*_ = 10 %

The image *L*_*V*_(*m*, *n*) contains a lot of artefacts resulting from the noise occurring in the border images *L*_*V*_(*m*, *n*, *i*) for *i* ∈ {1, 2, *I* *−* 1, *I*}. Therefore, 3D filtration was proposed, which enabled median filtering of each pixel for the 8-neighbourhood system. Schematic diagram of the filtration process for a three-dimensional plane of an object (border) is shown in Fig. 4a. The process of filtration relates to the 8-neighbourhood system of each pixel of the matrix *L*_*v*_(*m*, *n*, *i*) on the basis of which the median value is calculated. A median filter is used in relation to the plane formed from the object—in this case sized 30 × 30 pixels (see Fig. 4a). On this basis, the filtered image *Ls*(*m*, *n*), after binarization with the thresholds *p*_*r1*_*, p*_*r2*_ and *p*_*r3*_ equal to 30, 20 and 10 % respectively (thresholding with the upper threshold), is shown using colours (red, green and blue respectively) in Fig. 4b. This image, *L*_*S*_(*m*, *n*), resulting from binarization with the properly selected (manually or automatically) threshold, is the result of segmentation.

As it can be concluded from the above description, due to the specificity of this segmentation method, the time of analysis is at the level of 1949 ms for the processor Intel^®^ Core i7 4960X CPU 3.6 GHz.

### Hierarchical segmentation

**Fig. 5:**
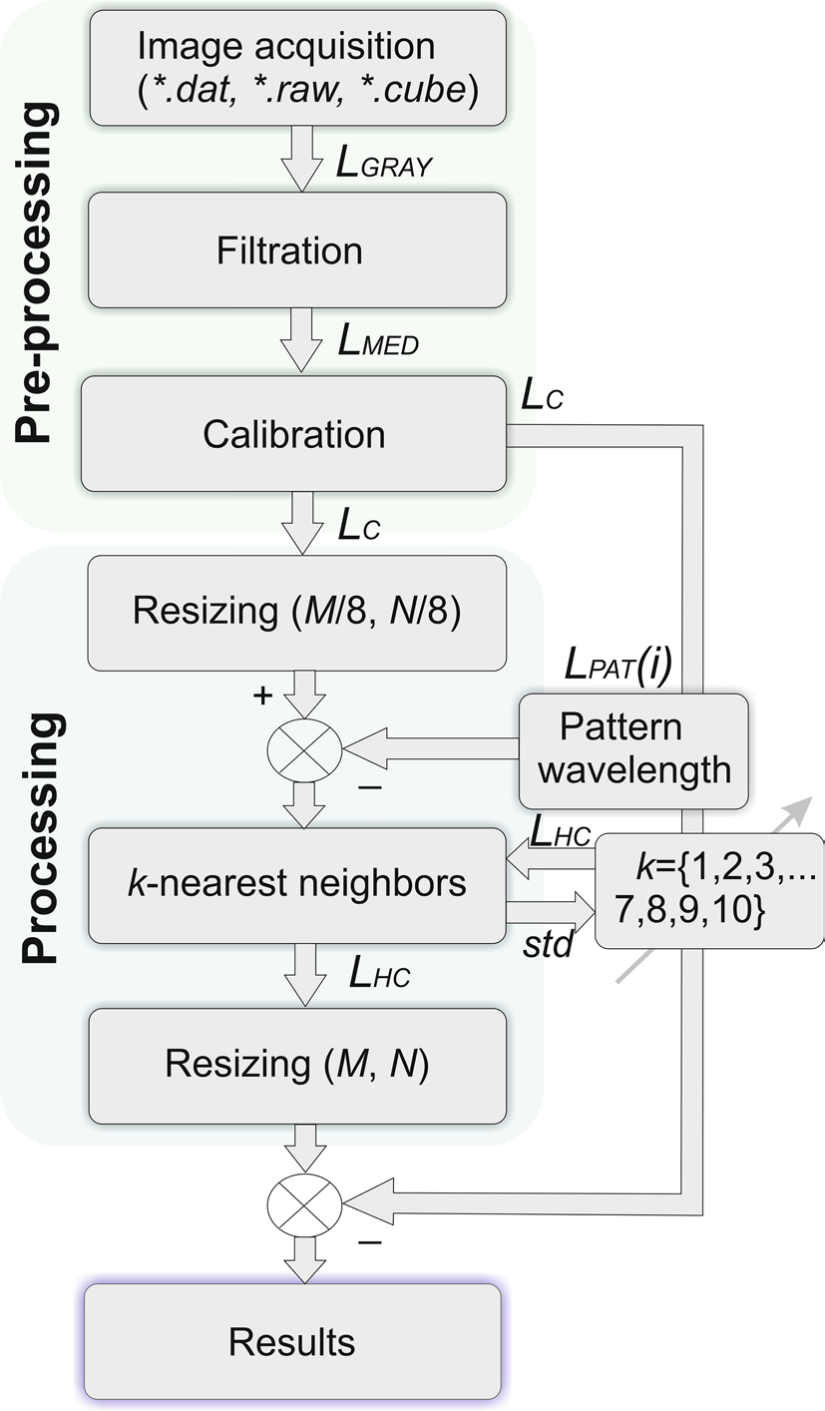
Simplified block diagram of the proposed hierarchical segmentation method

The dedicated hierarchical method gives the best results in segmentation. The word hierarchical refers here to the hierarchy in the resolution analysis of the image *L*_*C*_(*m*, *n*, *i*). The analysis is associated with the analysis of the resolution of the image *L*_*C*_(*m*, *n*, *i*) reduced to *M*/8 × *N*/8. Then, the analysis of the *k*-nearest neighbours was proposed. The *k*-nearest neighbour analysis was implemented for three features, *w*(1), *w*(2) and *w*(3). These features are absolute values of differences between the analysed image *L*_*C*_(*m*, *n*, *i*) and the reference waveform *L*_*PAT*_(*i*) for the selected three wavelengths. The three selected wavelengths are, in this case, the local maxima of the waveform *L*_*PAT*_(*i*). In general, the number of features [local maxima of the waveform *L*_*PAT*_(*i*)] is arbitrary. It can be limited only by computational complexity (the need to determine individual local maxima). In this example, due to the facilitated visualization (3D graph), the number of sought local maxima was limited to three [three features *w*(1), *w*(2) and *w*(3)]. Therefore, the segmentation method starts with classification in 2, 3, 4 and 5 regions, taking into account the features *w*(1), *w*(2) and *w*(3)—Fig. 5.

**Fig. 6:**
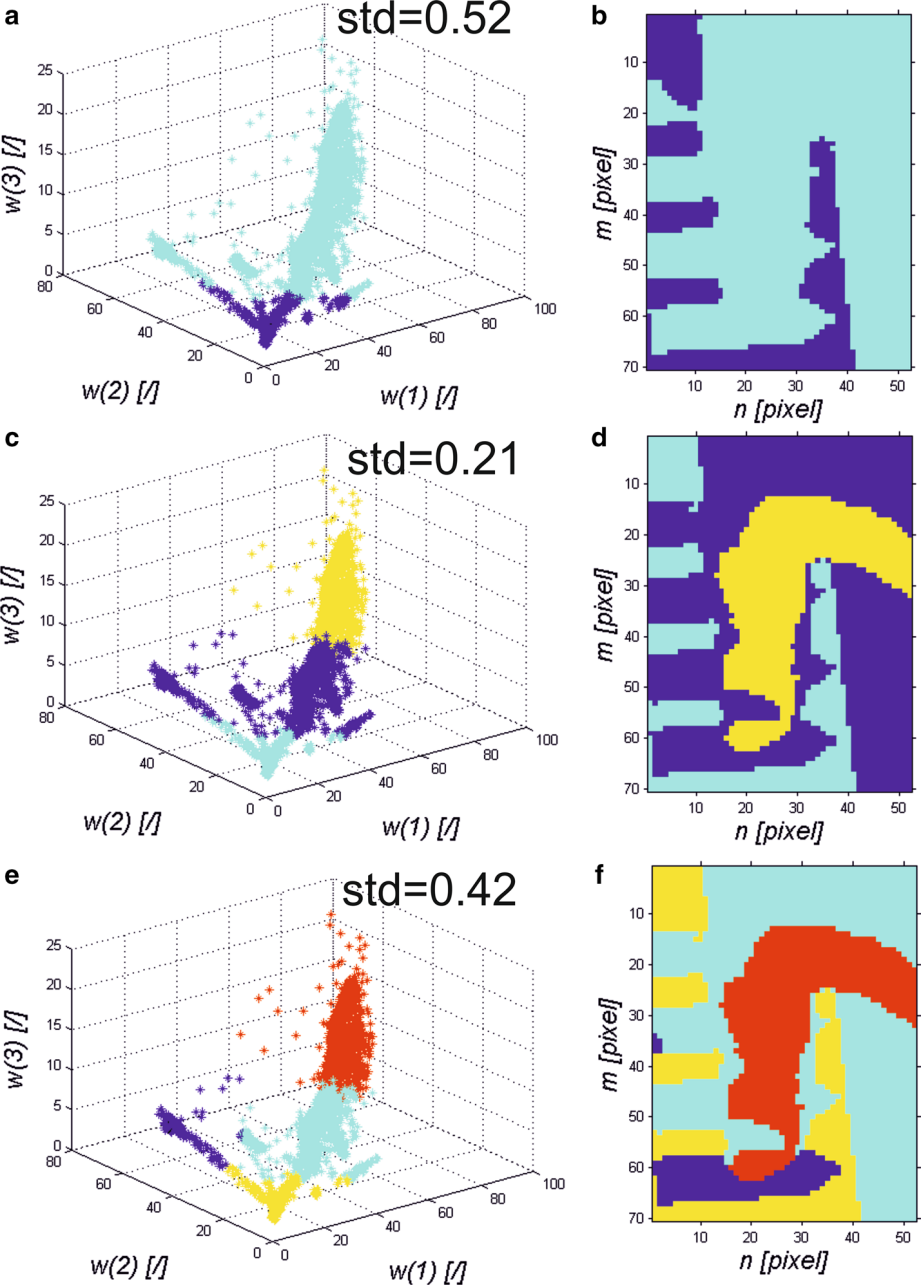
Sample result of segmentation based on the analysis of the nearest neighbours: **a**, **c**, **e** graphs of changes in the value for the next features *w*(1), *w*(2) and *w*(3) and **b**, **d**, **f** results of segmentation for 2, 3 and 4 classes respectively

As the number of regions, the number of classes, is not generally known (the number of the segmented objects), the value of standard deviation of the mean (*std*) is used as a criterion. The number of classes is increased from 2 to 5 regions. The correct number of classes is the one for which the value of the inter-class mean square error (*std*) is the smallest. Figure 6 shows examples of segmentation results for the thumb (Fig. 2a). The results were obtained for two, three and four classes and three features [*w*(1), *w*(2) and *w*(3)].

For the sample results shown in Fig. 6, the smallest value of the mean square error (*std* = 0.21) was for 3 classes. The image *L*_*HC*_(*m*, *n*, *i*) created in this way is the basis for increasing its resolution and automatic designation of segmented areas in the source image *L*_*GRAY*_(*m*, *n*, *i*) or in the image after pre-processing of the images *L*_*C*_(*m*, *n*, *i*) (Fig. 5).

**Fig. 7:**
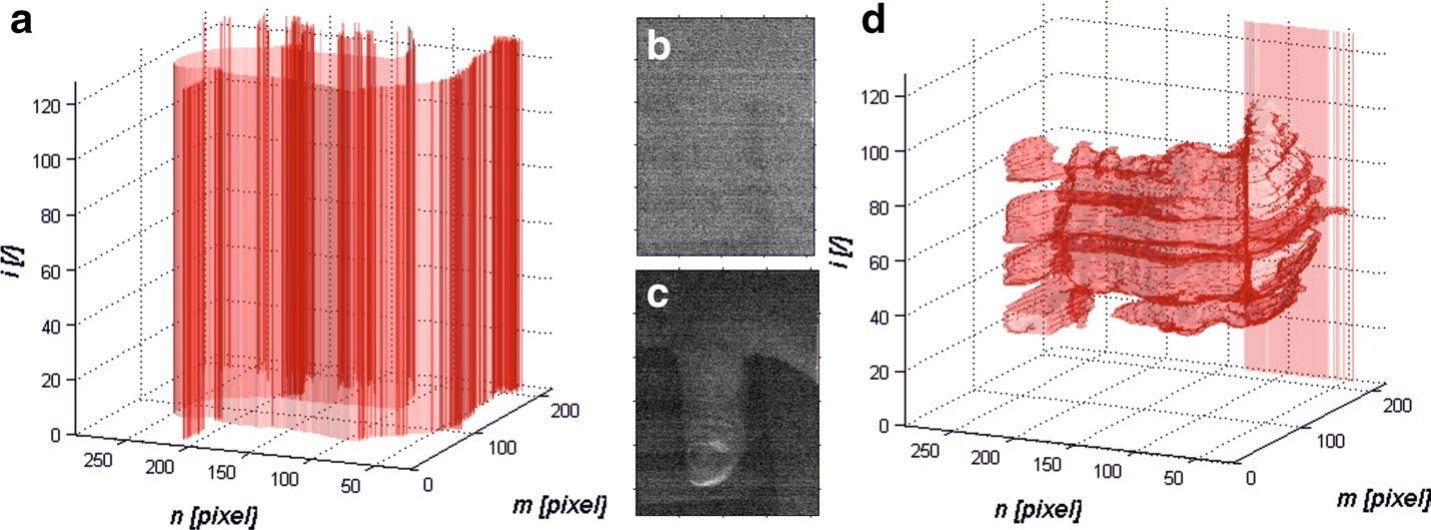
Sample segmentation of the objects without and with erosion and conditional dilatation: **a** the results of segmentation in the case of no erosion and conditional dilatation—e.g. as a result of a simplified method: **b**, **c** sample images for extreme values *i* = 10 and *i* = 110 showing the level of noise for extreme wavelengths and **d** the result of segmentation of the same object with the use of erosion and conditional dilatation

Hyperspectral images segmentation [27–29] with the hierarchical method is linked with yet another new question concerning the variability of size/shape of the object in the segmentation for next *i*-th values taking into consideration the criterion described with the formula (4). The segmented object may therefore change its shape fulfilling the criterion (4) for the next *i*-th images. A simple method of binarization conducted for the next *i*-th images does not render satisfying results due to the possibility of adding next new objects (not by changing the size of the existing segmented object). Therefore, erosion *L*_*E*_(*m*, *n*, *i*) was proposed with conditional dilatation *L*_*D*_(*m*, *n*, *i*) performed for each *i*-th binary image starting from *L*_*BIN*_(*m*, *n*, *i* = *1*), i.e.:

7 $$L_{E}^{ } \left( {m,\;n,\;i = 1} \right) = \left\{ {\begin{array}{*{20}l} {L_{BIN}^{ } \left( {m,\;n,\;i = 1} \right)} &\quad {\text{if}}\, {p_{c} \left( {m,\;n,\;i = 1} \right) < p_{dc} } \\ {\mathop {\hbox{min} }\limits_{{m_{S} ,n_{S} \in SE}} \left( {L_{BIN}^{ } \left( {m + m_{S} ,n + n_{S} ,i = 1} \right)} \right)} &\quad {\text{other}} & \\ \end{array} } \right.$$

8 $$L_{D}^{ } \left( {m,\;n,\;i = 1} \right) = \left\{ {\begin{array}{*{20}l} {L_{BIN}^{ } \left( {m,\;n,\;n = 1} \right)} &\quad {\text{if}} \,{p_{c} \left( {m,\;n,\;i = 1} \right) > p_{ec} } \\ {\mathop {\hbox{max} }\limits_{{m_{S} ,n_{S} \in SE}} \left( {L_{BIN}^{ } \left( {m + m_{S} ,n + n_{S} ,n = i} \right)} \right)} &\quad {\text{other}} & \\ \end{array} } \right.$$

where:

9 $$p_{c} \left( {m,\;n,\;i} \right) = \frac{1}{{M_{S} \cdot N_{S} }}\mathop \sum \limits_{{m_{S} = 1}}^{{M_{S} }} \mathop \sum \limits_{{n_{S} = 1}}^{{N_{S} }} L_{GRAY}^{ } \left( {m + m_{S} ,\;n + n_{S} ,\;i} \right)$$

and *m*_*S*_, *n*_*S*_ the row and column position of the mask *SE* sized *M*_*S*_ × *N*_*S*_ depending on the size of the segmented object. The mask changed in this case has a size of *M*_*S*_ × *N*_*S*_ = 17 × 17 pixels. The values of thresholds *p*_*ec*_ and *p*_*dc*_ are set by the user once for all images from the given type of camera. In the present case of SOC710 Hyperspectral Imaging System the value was *p*_*ec*_ = *p*_*dc*_ = 0.5. The results for the segmentation without erosion and conditional dilatation and with erosion and conditional dilatation are shown in Fig. 7.

## Experimental and discussion

The three new proposed segmentation methods need to be tested in practice in order to confirm the accuracy and time of analysis. The basis for the performed experiments is:

- The three methods described in this article:
  - fast analysis of emissivity curves (SKE),
  - 3D segmentation (S3D),
  - hierarchical segmentation (SH),
- The three known segmentation methods (for quantitative comparison of the results obtained):
  - method based on brightness thresholding (SPJ)—the binarization threshold is selected manually and automatically using Otsu’s formulas [18],
  - watershed method (SWS) preceded by filtration with an averaging filter whose mask size is in the range from 3 × 3 pixels to 9 × 9 pixels,
  - method based on mathematical morphology (SMM), especially erosion and conditional dilation,
- Manual method of object selection considered further as the benchmark (SP).

Over 700 complete sequences of images *L*_*GRAY*_(*m*, *n*, *i*) (a total of more than 10,000 2D images), whose acquisition conditions are given in the “Materials” section, were subject to segmentation. Common measures were used to compare the quality of the detected objects—*FP* the number of false-positive detected pixels, *FN*—false negative as well as *TP*, *TN*—true negative and true positive respectively. On their basis, sensitivity *TPR* = *TP/*(*TP* + *FN*), specificity *SPC* = *TN*/(*TN* + *FP*) and balanced accuracy *ACC* = *(TPR* + *SPC)/*2 were determined. The obtained results of mean values of *TPR*, *SPC* and *ACC* for the compared segmentation methods are presented in Table 2.

**Table 2:** Comparison of the proposed dedicated methods of hyperspectral image segmentation with the known methods and the benchmark

	*TPR* (%)	*SPC* (%)	*ACC* (%)
SKE	78	79	79
S3D	88	92	90
SH	92	91	92
SPJ	77	82	80
SWS	76	79	77
SMM	66	90	78

According to the presented Table 2, segmentation based on a hierarchical approach, proposed in this article, has the greatest value of *ACC* (*ACC* = 92). The other proposed segmentation methods are at a similarly high level (of *ACC* values). Among the known non-profiled segmentation methods, the method based on brightness thresholding has the largest value of *ACC*. However, it is a semi-automatic method whose results are closely dependent on the method of selecting the binarization threshold. The other features of the discussed segmentation methods look slightly different—Table 3.

**Table 3:** Comparison of other features of the proposed dedicated and well-known methods of hyperspectral image segmentation

	Analysis time (ms)	Sensitivity of segmentation to image rotation (yes/no)	Dependence of the segmentation time on the number of objects	Application of emissivity curves
SKE	2.3	√	–	√
S3D	1949	–	–	√
SH	844	–	√	√
SPJ	About 2000 (manual method)	–	–	–
SWS	1800	√	√	–
SMM	3499	√	√	–

The results for a PC with Intel^®^ Core i7 4960X CPU 3.6 GHz clearly show the superiority of the method of 3D segmentation (S3D) whose analysis time does not depend on the number of detected (segmented) objects. Moreover, this method is not sensitive to image rotation. This is due to the idea of its operation shown in Fig. 4 where no direction is privileged. Of course, fast analysis of emissivity curves is the quickest—its analysis time for *m* = 1 and *i* = 1 is equal to 2.3 ms (Table 1). The other known segmentation methods consume several times more of the CPU time.

As it was mentioned in the “Background” section, numerous similar segmentation methods are known from the literature. Hence, the method utilizing emissivity curves of a given object (SKE method) is used in many applications such as specim spectral imaging [30, 31], hyperspectral imaging system [5, 18] or imageJ [32]. However, in none of such applications the time of preliminary segmentation was obtained taking into account the emissivity curve at the level of 2.3 ms. For example in [33] the authors obtain the time of analysis equal to 100 ms using Intel^®^ Core i5 CPU M460 @2.5 GHz 4 GB RAM. Usually, the time of analysis increases, e.g. in Specim systems, which results from the fact that the registered sequence of *i*-th 2D images is pre-processed. A significant element of this processing is lowering the resolution of images. Then, this initially-prepared 3D image is analyzed. In the case when many different objects are registered, the process becomes burdensome unnecessarily increasing the involvement of staff (operator). In the case of the second and third method in question (S3D and SH), the situation is different. These methods, especially SH based on erosion and conditional dilatation, is not used in any commercial applications concerning hyperspectral imaging and it was not presented in this use, namely for correction of hyperspectral images. The majority of authors, for instance in the articles [4, 6, 8, 11, 13], perform segmentation in the field of an image, one selected 2D image from *i*-th images of a sequence. Another widely tested approach is the use of clustering in the space of particular wavelengths e.g. at work [6]. Both mentioned types of segmentation do not cope with two elements: variable levels of noise of hyperspectral images for band extreme values and with the correction of the shape of the segmented image for subsequent *i*-th images. In particular, the correction of the object shape for subsequent *i*-th 2D images of the series is especially important in terms of determining the compatibility of its emissivity curves with the calibration curve. Therefore, the methods presented in this paper are new segmentation methods which provide a new quality in a considerably shorter period of time when compared to the known methods of hyperspectral image segmentation.

## Conclusion

The presented three new segmentation methods have the following advantages:

- they are fully automatic,
- they enable to use a fast segmentation method
- they are profiled to hyperspectral image segmentation,
- they use emissivity curves as the model,
- they can be applied to any type of objects (not necessarily biological),
- they are faster and more precise compared to conventional methods known from the literature.

Work is currently underway to implement the described segmentation methods, especially the first described fast method for digital circuits DSP. The aim is the initial, at the acquisition phase, object segmentation. This will enable to automatically limit the acquired image and, thus, reduce the hyperspectral camera operation time.

## Abbreviations

ROI: region of interest
SKE: fast analysis of emissivity curves
S3D: segmentation 3D
SH: hierarchical segmentation
SPJ: method based on brightness thresholding
SWS: watershed method
SMM: method based on mathematical morphology
SP: manual method
